# ABCB1 Polymorphism in HIV‐Infected Individuals Taking Antiretroviral Drugs

**DOI:** 10.1155/arat/9656615

**Published:** 2025-10-16

**Authors:** HariOm Singh, Dharmesh Samani, Supriya D. Mahajan

**Affiliations:** ^1^ Department of Molecular Biology, ICMR-National AIDS Research Institute, Pune, 411026, India; ^2^ Department of Medicine, Jacobs School of Medicine & Biomedical Sciences, University at Buffalo’s Clinical Translational Research Center, 875 Ellicott Street, Buffalo, New York, 14203, USA

**Keywords:** ABC transporter P-glycoprotein, ARV-associated hepatotoxicity, genetic polymorphisms, haplotype, HIV

## Abstract

ABC transporter P‐glycoprotein (P‐gp) and its expression enhance elimination and reduce drug exposure. The elimination of non‐nucleoside reverse‐transcriptase inhibitor (NNRTIs) drugs is associated with *ABCB1* gene. Drug exposure is impacted by variants in the ABCB1 gene. Hence, the aim of the study was to investigate the association of *ABCB1* 1236 C/T and 3435 C/T polymorphisms with the modulation of antiretroviral (ARV)‐associated hepatotoxicity. This is a cross‐sectional study. Genotyping of the ABCB1 1236C/T and 3435C/T polymorphisms was performed in 165 HIV‐infected individuals (34 with hepatotoxicity and 131 without hepatotoxicity) and 155 healthy controls using the PCR‐RFLP method. The TC haplotype was likely to be associated with a higher risk of severe hepatotoxicity (OR = 1.96, *p* = 0.06), while CC and TT haplotypes were associated with a reduced risk of severe hepatotoxicity (OR = 0.34, *p* = 0.039; OR = 0.16, *p* = 0.006; OR = 0.09, *p* = 0.003). The *ABCB1* 3435CT genotype along with alcohol usage revealed a risk of HIV disease progression (OR = 2.47, *p* = 0.12). The *ABCB1* 1236TT genotype along with nevirapine (NVP) usage exhibited a risk for hepatotoxicity severity (OR = 2.11, *p* = 0.55). The *ABCB1* 3435CT genotype along with alcohol + NVP usage bared a risk of acquisition of hepatotoxicity (OR = 2.04, *p* = 0.23). In conclusion, ABCB1 haplotypes may influence the severity of ARV‐induced hepatotoxicity. While individual polymorphisms and their interaction with alcohol or drug regimen showed no significant association, the 1236TT genotype with NVP use and the TC haplotype suggested a potential risk. Protective effects were observed for CC and TT haplotypes. Larger studies are warranted to confirm these findings and assess clinical relevance.

## 1. Introduction

Antiretroviral (ARV) treatment is commonly used to treat human immunodeficiency virus (HIV) patients. However, the selection of an antiretroviral therapy (ART) regimen is the main issue in treating HIV infection because of its efficacy and toxicity. Higher plasma concentrations of ARV drugs in HIV‐positive patients are associated with adverse drug reactions (ADRs), including hepatotoxicity. A nevirapine (NVP)‐based regimen shows a higher incidence of hepatotoxicity compared to an efavirenz (EFV)‐based regimen [1]. Hepatotoxicity was seen in 3.11% of NVP users [2]. Therapy with NVP is associated with significant elevations of the alanine transaminase (ALT) level in 4%–20% of patients and symptomatic ALT level increases in 1%–5% of patients [3]. Hepatotoxicity severity was 10.8% in the group treated with EFV and 8.9% in the group treated with NVP [4]. The transporter (*ABCB1*) gene affects the oral absorption and tissue penetration of non‐nucleoside reverse‐transcriptase inhibitors (NNRTIs), EFV, and NVP [5, 6] and is involved in the regulation of cell homeostasis [7].

The *ABCB1* gene encodes P‐glycoprotein (P‐gp), a transmembrane transporter protein. P‐gp is an ATP‐dependent efflux system that transports substances, including drugs and nucleosides, from the intracellular to the extracellular matrix [8]. It is crucial for the uptake, distribution, and excretion of a variety of substrates and drugs [9]. The *ABCB1* gene is expressed in several organs, including blood–brain barrier endothelial cells [10]. Genetic variations in ABC transporters play a significant role in drug resistance, ADRs, and treatment outcomes [11]. The differential expression of P‐gp could impact the transportation, absorption, and penetration of NVP and EFV [12].

The *ABCB1* gene is located on Chromosome 7q21 [13]. *ABCB1* has more than 50 exonic single‐nucleotide polymorphisms (SNPs) [14], among which the frequencies of SNPs 1236 C/T, 2677 G/T/A, and 3435 C/T are varied (> 0.1) in various groups [15, 16]. Two haplotypes, *ABCB1*∗1 (1236C‐2677G‐3435C) and *ABCB1*∗13 (1236T‐2677T‐3435T), are commonly identified in various populations [17]. The *ABCB1* 3435 C/T polymorphism affects its expression level [18]. The *ABCB1* 3435 C/T polymorphism influences variations in substrate specificity; however, the molecular mechanism behind this effect is poorly understood [8].

The prevalence of the *ABCB1* 3435CC genotype was 85.9% in Africans, 41.70% in Indians, and 35.7% in White people in Kwazulu‐Natal and South Africa [19]. Patients with the *ABCB1* 3435CC genotype were correlated with P‐gp overexpression in the African population, whereas patients with genotype 3435TT had a decreased expression of P‐gp [20].

*ABCB1* 3435 C/T polymorphism was not associated with exposure to EFV [21]. Ritchie et al. demonstrated a significantly reduced incidence of hepatotoxicity in patients undergoing NNRTI therapy when an *ABCB1* 3435 C/T polymorphism was present [22]. Studies have also shown a link between the *ABCB1* 3435 C/T polymorphism and hepatotoxicity caused by NVP [22, 23]. *The ABCB1* 1236 C/T and 1236 C/T polymorphisms are linked to high EFV concentrations [24]. The *ABCB1* 1236 C/T and 3435 C/T polymorphisms and plasma dolutegravir levels are not correlated [25].

There are minimal studies regarding an association between *ABCB1* polymorphisms and adverse effects of ARV drugs, and the results are inconclusive. Nevertheless, no data associating the *ABCB1* polymorphism with ARV‐induced hepatotoxicity from India exist. Therefore, we examined the association of *ABCB1* polymorphisms (1236 C/T and 3435 C/T) with the susceptibility to the acquisition of ARV‐associated hepatotoxicity *and its severity*.

## 2. Methods

### 2.1. Study Design

The present study is a cross‐sectional, case–control genetic association study from November 2012 to February 2015. One hundred and sixty‐five HIV‐infected individuals underwent a liver function test (LFT). Out of that, 34 were HIV‐infected individuals with hepatotoxicity (Grade III or IV) under an NNRTI‐containing ART regimen, 131 were without hepatotoxicity, and they were age‐matched with 155 healthy individuals. These were taken consecutively from outpatient clinics at the National AIDS Research Institute, Pune. In the group of HIV‐infected individuals with hepatotoxicity, individuals with Hepatitis B, Hepatitis C, tuberculosis, concurrent untreated opportunistic infections, immune reconstitution syndrome, and any other known hepatotoxic drugs were excluded from the cases. At the same time, 155 individuals (excluded individuals from the same family) who were without HIV and Hepatitis B and C, tuberculosis free, age‐matched, and serum‐negative from the HIV‐ELISA test were recruited and described as healthy controls. We obtained clinical data through questionnaires, personal interviews, and the review of case records. The medical experts performed an LFT to assess the status of liver enzymes. Total bilirubin > 3.22 mg/mL, serum glutamic oxaloacetic transaminase (SGOT) > 93.8 U/mL, serum glutamic pyruvic transaminase (SGPT) > 229.5 U/mL, and alkaline phosphatase > 550.8 U/mL for males with hepatotoxicity and total bilirubin > 3.22 mg/mL, SGOT > 163.2 U/mL, SGPT > 173.4 U/mL, and alkaline phosphatase > 550.8 U/mL for females with hepatotoxicity described as cases. HIV‐infected male and female individuals having total bilirubin < 1.24 mg/mL, SGOT < 32 U/mL, SGPT < 34 U/mL, and alkaline phosphatase < 108 U/mL were described as controls (https://doi.org/10.21203/rs.3.rs-1019782/v1).

We calculated the CD4 cell count using fluorescence‐activated cell sorting (FACS). CD4 status was used to classify patients into different subgroups. CD4 count < 200 cells/mm^3^ was defined as an advanced‐stage HIV, the range of 201–350 cells/mm^3^ was defined as an intermediate‐stage HIV, and count > 350 cells/mm^3^ was defined as an early stage HIV disease. ELISA for Hepatitis C and HBsAg testing was performed with the Ortho HCV‐ELISA test system (Ortho Clinical Diagnostics, Buckinghamshire, UK) and Murex HBsAg confirmatory Version 3 (DiaSorin, Dartford, UK) ELISA. The use of tobacco and alcohol was also recorded in the questionnaire. The study was approved by the ethics committee of the institute (ICMR‐NARI), and written informed consent was obtained from all the recruited participants.

### 2.2. Extraction of DNA

Two milliliter of peripheral blood was drawn from subjects and stored at −70°C until DNA extraction. DNA extraction was carried out from peripheral blood leukocyte pellets using the QIAamp DNA Mini Kit (QIAGEN Str. 1 40724 Hilden, Germany) according to the manufacturer’s guidelines.

### 2.3. Genotyping

The *ABCB1* (1236 C/T and 3435 C/T) polymorphisms were genotyped in subjects using PCR–restriction fragment length polymorphism (PCR–RFLP), and amplification primers for *ABCB1* (1236 C/T and 3435 C/T) were adopted from an earlier report [26–28]. PCR was performed in a total volume of 25 μL with 10 pmol of each primer, genomic DNA (100—150 ng), 2.5 mM deoxynucleotide triphosphates, PCR buffer containing 100 mM Tris–HCl, pH 8.6, 50 mM KCl, 1.5 mM MgCl_2_, and 1 unit of Taq polymerase (New England Biolabs, USA). The reaction conditions for *ABCB1* 1236 C/T were as follows: initial denaturation at 95°C for 5 min, followed by 40 cycles of denaturation at 95°C for 30 s, annealing at 58°C for 30 s, extension at 72°C for 45 s, and a final extension at 72°C for 10 min. The conditions for *ABCB1* 3435 C/T were as follows: initial denaturation at 95°C for 2 min, followed by 35 cycles of denaturation at 95°C for 30 s, annealing at 60°C for 1 min, extension at 72°C for 45 s, and a final extension at 72°C for 10 min. All reactions were carried out in a Veriti 96‐well thermal cycler (Applied Biosystems, CA, USA). PCR products and molecular weight markers were visualized by ethidium bromide staining. *ABCB1* 1236 C/T and 3435 C/T amplified products were digested using restriction enzymes at 37°C for 16 h using *Hae*III and *Mbo*I, respectively (MBI Fermentas Inc., Hanover, MD, USA). Genotyping of *ABCB1* 1236 C/T and 3435 C/T was done on a 10% polyacrylamide gel using molecular weight markers and visualized after staining with ethidium bromide. Based on sequences and location of SNP, genotypes of *ABCB1* 1236 C/T and 3535 C/T were assigned as follows: for *ABCB1* 1236 C/T: 93 and 87 bp for CC; 87, 58 and 35 bp for TT; and 93, 87, 58, and 35 bp for CT, whereas those of *ABCB1* 3435 C/T were assigned as follows: 130 and 76 bp for CC; 206 bp for TT; and 206, 130, and 76 bp for CT. Re‐genotyping in 20% of the samples was done by other laboratory personnel, and no discrepancies in genotyping were noticed. Ten per cent of the samples were sequenced to assess the genotyping error.

### 2.4. Statistical Examination

The age variable was expressed as mean ± standard deviation (SD). The *χ* ^2^ goodness‐of‐fit test was used for any deviation from Hardy–Weinberg equilibrium in the controls. We used the *χ* ^2^ statistic (Fisher’s exact test for cell size < 5) to compare the genotype frequency between HIV‐infected individuals with hepatotoxicity versus those without hepatotoxicity and HIV‐infected individuals versus healthy controls. The SNPStats software tool was used to compare the haplotype frequency between HIV‐infected individuals with hepatotoxicity versus those without hepatotoxicity, HIV‐infected individuals with hepatotoxicity versus healthy controls, and HIV‐infected individuals without hepatotoxicity versus healthy controls [29]. The unconditional binary logistic regression was used to calculate odds ratios (ORs) and 95% confidence intervals (CIs). All other statistical analyses were performed using SPSS software, Version 17.0 (SPSS, Chicago, IL, USA), and tests of statistical significance were two‐sided, and a *p* value less than 0.05 was considered significant. LD was assessed between both loci by calculating the relative LD value (D′) as D′ = Dij/Dmax [30]. The Dij values were compared between HIV‐infected individuals with hepatotoxicity versus those without hepatotoxicity, HIV‐infected individuals with hepatotoxicity versus healthy controls, and HIV‐infected individuals without hepatotoxicity versus healthy controls by comparison with CIs.

## 3. Results

### 3.1. General Characteristics of the Study Population

The study population included 34 HIV‐infected individuals with hepatotoxicity, 131 without hepatotoxicity, and 155 healthy controls. The mean ages ± SD of HIV‐infected individuals with hepatotoxicity, without hepatotoxicity, and healthy controls were 35.87 ± 8.96, 39.29 ± 1.34, and 27.74 ± 8.50 years, respectively. Table 1 summarizes the characteristics of all study participants.

**Table 1:** General characteristic of the study population.

Subjects	HIV‐infected individuals with hepatotoxicity (Grades III and IV)	HIV‐infected individuals without hepatotoxicity	Healthy controls
Number	*N* = 34	*N* = 131	*N* = 155
Mean age (range)	35.14 ± 8.96	39.29 ± 1.34	36.75 ± 8.50
Females	16 (47.05)	47 (35.87)	43 (27.74)
Males	18 (52.94)	84 (64.12)	112 (72.25)

*NNRTI regimen*			
Efavirenz *N* = 23	11 (32.35)	12 (9.16)	Not applicable (NA)
Nevirapine *N* = 142	23 (67.64)	119 (90.83)	Not applicable

*Alcohol habit*			
User *N* = 51	7 (20.58)	44 (33.58)	0
Nonuser *N* = 114	27 (79.41)	87 (66.41)	0

*Tobacco habit*			
User *N* = 50	23 (67.64)	27 (20.61)	0
Nonuser *N* = 115	11 (32.35)	104 (79.38)	0

*CD4+ status*			
< 200 (*N* = 95)	16 (47.05)	41 (31.29)	NA
201–350 (*N* = 50)	17 (50)	33 (25.19)	NA
> 350 (*N* = 20)	1 (2.94)	19 (14.50)	NA

*Note: N*, total number of subject participants; 0, alcohol, and tobacco status was not reported.

Abbreviations: NA, not applicable; NNRTI, non‐nucleoside reverse‐transcriptase inhibitors.

### 3.2. Genotypic and Allelic Distribution of ABCB1 Polymorphisms

Table 2 presents the genotype and allele frequency of ABCB1 (1236 C/T and 3435 C/T) polymorphisms. No significant differences in ABCB1 polymorphisms were observed between HIV‐infected individuals with and without hepatotoxicity. For instance, the 1236TT genotype was observed in 17.6% and 12.2% of individuals with and without hepatotoxicity, respectively (OR = 1.38, 95% CI: 0.45–4.12, *p* = 0.57). Similarly, the 3435TT genotype was found in 35.3% of individuals with hepatotoxicity and 43.5% without (OR = 0.56, 95% CI: 0.20–1.59, *p* = 0.28).

**Table 2:** Genotypic and allelic distribution of ABCB1 polymorphisms in the study population.

**Genotype *ABCB1* (1236** **C/T)**	**HIV-infected individuals with hepatotoxicity *N* = 34 (%)**	**HIV-infected individuals without hepatotoxicity *N* = 131 (%)**	**p** **value**	**OR (95% CI)**
CC	16 (47.1%)	59 (45.0%)		1 (Reference)
CT	12 (35.3%)	56 (42.7%)	0.37	0.68 (0.29–1.60)
TT	6 (17.6%)	16 (12.2%)	0.57	1.37 (0.45–4.12)
CT + TT	18 (52.94)	72 (54.96)	0.63	0.82 (0.38–1.79)
***ABCB1* (1236** **C/T) allele**	**HIV-infected individuals with hepatotoxicity *N* = 68 (%)**	**HIV-infected individuals without hepatotoxicity *N* = 262 (%)**	**p** **value**	**OR (95% CI)**
C	44 (64.71%)	174 (66.41%)	—	1 (Reference)
T	24 (35.29%)	88 (33.59%)	0.90	1.08 (0.59–1.95)
**Genotype *ABCB1* (3435** **C/T)**	**HIV-infected individuals with hepatotoxicity *N* = 34 (%)**	**HIV-infected individuals without hepatotoxicity *N* = 131 (%)**	**p** **value**	**OR (95% CI)**
CC	8 (23.5%)	24 (18.3%)	—	1 (Reference)
CT	14 (41.2%)	50 (38.2%)	0.70	0.82 (0.30–2.25)
TT	12 (35.3%)	57 (43.5%)	0.28	0.56 (0.20–1.59)
CT + TT	26 (74.47%)	107 (81.67%)	0.42	0.68 (0.27–1.71)
***ABCB1* (3435** **C/T) allele**	**HIV-infected individuals with hepatotoxicity *N* = 68 (%)**	**HIV-infected individuals without hepatotoxicity *N* = 262 (%)**	**p** **value**	**OR (95% CI)**
C	30 (44.12%)	98 (37.40%)	—	1 (Reference)
T	38 (55.88%)	164 (62.59%)	0.38	0.76 (0.43–1.35)
**Genotype *ABCB1* (1236 C/T)**	**HIV-infected patients with hepatotoxicity *N* = 34 (%)**	**Healthy control *N* = 155 (%)**	**p** **value**	**OR (95% CI)**
CC	16 (47.1%)	69 (44.52%)		1 (Reference)
CT	12 (35.3%)	65 (41.94%)	0.45	0.70 (0.27–1.79)
TT	6 (17.6%)	21 (13.54%)	0.98	0.99 (0.29–3.38)
CT + TT	18 (52.94)	86 (55.48)	0.55	0.77 (0.33–1.82)
***ABCB1* (1236 C/T) allele**	**HIV-infected individuals with hepatotoxicity *N* = 68 (%)**	**Healthy control *N* = 310**	**p** **value**	**OR (95% CI)**
C	44 (64.71%)	203 (65.48%)	—	1 (Reference)
T	24 (35.29%)	107 (34.52%)	0.98	1.03 (0.58–1.85)
**Genotype *ABCB1* (3435 C/T)**	**HIV-infected individuals with hepatotoxicity *N* = 34 (%)**	**Healthy control *N* = 155**	**p** **value**	**OR (95% CI)**
CC	8 (23.5%)	34 (21.94%)	—	1 (Reference)
CT	14 (41.2%)	67 (43.23%)	0.24	0.52 (0.17–1.58)
TT	12 (35.3%)	54 (34.83%)	0.28	0.53 (0.16–1.69)
CT + TT	26 (76.47)	121 (78.06)	0.22	0.52 (0.19–1.46)
***ABCB1* (3435 C/T) allele**	**HIV-infected individuals with hepatotoxicity *N* = 68 (%)**	**Healthy control *N* = 310**	**p** **value**	**OR (95% CI)**
C	30 (44.12%)	135 (43.54%)	—	1 (Reference)
T	38 (55.88%)	175 (56.45%)	0.96	0.98 (0.56–1.71)
**Genotype *ABCB1* (1236 C/T)**	**HIV-infected patients without hepatotoxicity *N* = 131 (%)**	**Healthy control *N* = 155 (%)**	**p** **value**	**OR (95% CI)**
CC	59 (45.0%)	69 (44.52%)	—	1 (Reference)
CT	56 (42.7%)	65 (41.94%)	0.65	0.87 (0.48–1.59)
TT	16 (12.2%)	21 (13.54%)	0.39	0.69 (0.29–1.61)
CT + TT	72 (54.96)	86 (55.48)	0.49	0.82 (0.47–1.43)
***ABCB1* (1236 C/T) allele**	**HIV-infected patients without hepatotoxicity *N* = 262 (%)**	**Healthy control *N* = 310**	**p** **value**	**OR (95% CI)**
C	174 (66.41%)	203 (65.48%)	—	1 (Reference)
T	88 (33.59%)	107 (34.52%)	0.88	0.96 (0.67–1.38)
**Genotype *ABCB1* (3435 C/T)**	**HIV-infected patients without hepatotoxicity *N* = 131 (%)**	**Healthy control *N* = 155**	**p** **value**	**OR (95% CI)**
CC	24 (18.3%)	34 (21.94%)	—	1 (Reference)
CT	50 (38.2%)	67 (43.23%)	0.53	0.79 (0.37–1.66)
TT	57 (43.5%)	54 (34.83%)	0.57	1.24 (0.59–2.61)
CT + TT	107 (81.67)	121 (78.06)	0.97	0.99 (0.50–1.94)
***ABCB1* (3435 C/T) allele**	**HIV-infected patients without hepatotoxicity *N* = 262 (%)**	**Healthy control *N* = 310**	**p** **value**	**OR (95% CI)**
C	98 (37.40%)	135 (43.54%)	—	1 (Reference)
T	164 (62.59%)	175 (56.45%)	0.16	1.29 (0.91–1.83)

*Note: N*, total number of HIV‐infected individuals with hepatotoxicity (34), without hepatotoxicity (131), and healthy controls (155). Odds ratios and 95% CIs were derived from logistic regression models comparing the homozygous wild‐type genotype/allele (CC genotype and C allele were used as a reference for *ABCB1* 1236CT, 1236TT genotypes, and 1236 T allele and 3435CT, 3435TT genotypes, and 3435T allele) with other genotypes/alleles.

Both SNPs followed Hardy–Weinberg equilibrium in the control group. While most genotype and allele frequencies were similar across all groups, the 3435TT genotype was slightly more prevalent in HIV‐infected individuals than in healthy controls (43.5% vs. 34.83%).

Both SNPs conformed to Hardy–Weinberg equilibrium in the control group. While the 3435TT genotype appeared slightly more frequent in HIV‐infected individuals than in healthy controls (43.5% vs. 34.8%), these differences were not statistically significant. Given the wide CIs and *p* values > 0.05, the results indicate weak evidence of association and should be interpreted cautiously.

### 3.3. Haplotype Frequencies Between HIV‐Infected Individuals With and Without Hepatotoxicity

Table 3 shows the distribution of ABCB1 haplotypes. Using CT (1236C/3435T) as the reference, the TT haplotype was associated with reduced odds of hepatotoxicity severity (0.05% vs. 0.22%, OR = 0.16, 95% CI: 0.04–0.59, *p* = 0.0065), suggesting a potential protective association. The TC haplotype showed a higher frequency in the hepatotoxicity group compared to those without (0.30% vs. 0.11%, OR = 1.96, 95% CI: 0.98–3.94, *p* = 0.06). However, due to the borderline *p* values and wide CIs, these findings should be viewed as preliminary and require validation in larger, well‐powered cohorts.

**Table 3:** Frequency distribution of haplotype *ABCB1* (1236 C/T and 3435 C/T) in HIV‐infected individuals with and without hepatotoxicity and healthy controls.

Haplotype *ABCB1* (1236 C/T and 3435 C/T)	HIV‐infected individuals with hepatotoxicity (*N* = 68)	HIV‐infected individuals without hepatotoxicity (*N* = 262)	*p* value	OR (95% CI)
CT	0.51	0.41	—	1 (Reference)
CC	0.14	0.26	0.062	0.46 (0.20–1.03)
TT	0.05	0.22	**0.006**	0.16 (0.04–0.59)
TC	0.30	0.11	**0.06**	1.96 (0.98–3.94)

**Haplotype *ABCB1* (1236 C/T and 3435 C/T)**	**HIV-infected individuals with hepatotoxicity (*N* = 68)**	**Healthy controls (*N* = 310)**	**p** **value**	**OR (95% CI)**

CT	0.51	0.35	—	1 (Reference)
CC	0.14	0.30	**0.03**	0.34 (0.12–0.94)
TT	0.05	0.22	**0.003**	0.09 (0.02–0.44)
TC	0.30	0.13	0.11	1.94 (0.87–4.37)

**Haplotype *ABCB1* (1236 C/T and 3435 C/T)**	**HIV-infected individuals without hepatotoxicity (*N* = 262)**	**Healthy controls (*N* = 310)**	**p** **value**	**OR (95% CI)**

CT	0.41	0.35	—	1 (Reference)
CC	0.26	0.30	0.31	0.77 (0.46–1.28)
TT	0.22	0.22	0.31	0.75 (0.43–1.31)
TC	0.11	0.13	0.43	0.77 (0.40–1.48)

*Note: N*, total number of alleles in HIV‐infected individuals with hepatotoxicity (68), without hepatotoxicity (262), and healthy controls (310). OR and 95% CIs were derived from the logistic regression model comparing the haplotype CT (as a reference) with other haplotypes. Significant *p* values are shown in bold (*p* < 0.05).

### 3.4. ABCB1 Polymorphisms Across HIV Disease Stages

Table 4 summarizes the genotype distributions across different HIV disease stages and controls. No statistically significant differences were found, although 1236CT and 3435TT genotypes appeared more frequently in early stage HIV‐infected individuals compared to healthy controls. These nonsignificant observations may point to potential trends that require confirmation in longitudinal studies.

**Table 4:** ABCB1 polymorphism distribution in HIV‐infected individuals at different stages.

Genotype *ABCB1* (1236 C/T)	Healthy controls *N* = 155 (%)	Early stage of HIV infection		Intermediate stage of HIV infection		Advanced stage of HIV infection	
		*N* = 17 (%)	OR (*p*)	*N* = 52 (%)	OR (*p*)	*N* = 96 (%)	OR (*p*)
CC	69 (44.52%)	6 (35.3%)	1 (Reference)	27 (51.9%)	1 (Reference)	42 (45.8%)	1 (Reference)
CT	65 (41.94%)	9 (52.9%)	1.59 (0.56)	15 (51.9%)	0.59 (0.20)	44 (45.8%)	1.11 (0.80)
TT	21 (13.54%)	2 (11.8%)	1.10 (0.74)	10 (19.2%)	1.22 (0.83)	10 (10.4%)	0.78 (0.71)

**Genotype *ABCB1* (3435 C/T)**	**Healthy controls *N* = 155 (%)**	**Early stage of HIV**		**Intermediate stage of HIV**		**Advanced stage of HIV**	
		***N* = 17 (%)**	**OR (** **p** **)**	***N* = 52 (%)**	**OR (** **p** **)**	***N* = 96 (%)**	**OR (** **p** **)**

CC	34 (21.94%)	3 (17.6%)	1 (Reference)	10 (19.2%)	1 (Reference)	19 (19.8%)	1 (Reference)
CT	67 (43.23%)	6 (35.3%)	1.01 (0.72)	21 (40.4%)	1.07 (0.94)	37 (38.5%)	0.99 (0.88)
TT	54 (34.83%)	8 (47.1%)	1.68 (0.68)	21 (40.4%)	1.32 (0.67)	40 (41.7%)	1.33 (0.53)

*Note: N* = number of subjects, % = frequency of subjects, OR, and 95% CIs were derived from the logistic regression model comparing the homozygous wild‐type genotype/allele (CC genotype and C allele were used as a reference for *ABCB1* 1236CT, 1236TT genotypes and 1236 T allele and 3435CT, 3435TT genotypes and 3435T allele) with other genotypes/alleles.

### 3.5. ABCB1 Polymorphisms in Relation to Tobacco, Alcohol, and NNRTI Use

Table 5 provides the stratified analyses based on tobacco, alcohol, and NNRTI usage. While ABCB1 polymorphism frequencies were not significantly different across lifestyle factors or NNRTI regimens, some trends were observed:

• Smokers with hepatotoxicity had a higher frequency of 1236TT genotype than nonsmokers (28.6% vs. 14.8%, OR = 1.50, *p* = 0.88), though not statistically significant.
• Alcohol users with hepatotoxicity also had higher frequencies of 1236TT compared to nonusers (28.6% vs. 14.8%, OR = 1.50, *p* = 0.88).
• Among NNRTI users, NVP‐treated individuals showed a higher prevalence of 1236TT compared to EFV‐treated individuals (21.7% vs. 9.1%, OR = 2.11, *p* = 0.55), again without statistical significance.

**Table 5:** Frequency distribution of *ABCB1* (1236 C/T and 3435 C/T) genotypes in tobacco, alcohol, and NNRTI using HIV**-**infected individuals with hepatotoxicity and without hepatotoxicity.

**Genotype *ABCB1* (1236 C/T)**	**Tobacco users *N* = 7 (%)**	**Tobacco nonusers *N* = 27 (%)**	**p** **value**	**OR (95% CI)**
**HIV-infected individuals with hepatotoxicity**				
CC	4 (57.1%)	12 (44.4%)	—	1 (Reference)
CT	1 (14.3%)	11 (40.7%)	0.30	0.28 (0.024–3.18)
TT	2 (28.6%)	4 (14.8%)	0.88	1.50 (0.13–17.35)
**Genotype *ABCB1* (3435 C/T)**	**Tobacco users *N* = 7 (%)**	**Tobacco nonusers *N* = 27 (%)**	**p** **value**	**OR (95% CI)**
CC	3 (42.9%)	5 (18.5.0%)	—	1 (Reference)
CT	2 (28.6%)	12 (44.4%)	0.37	0.37 (0.042–3.25)
TT	2 (28.6%)	10 (37.0%)	0.59	0.53 (0.051–5.40)
**HIV-infected individuals without hepatotoxicity**				
**Genotype *ABCB1* (1236 C/T)**	**Tobacco users *N* = 43 (%)**	**Tobacco nonuser *N* = 88 (%)**	**p** **value**	**OR (95% CI)**
CC	19 (44.2%)	40 (45.5%)	—	1 (Reference)
CT	19 (44.2%)	37 (42.0%)	0.44	1.39 (0.60–3.20)
TT	5 (11.6%)	11 (12.5%)	0.90	1.08 (0.31–3.75)
**Genotype *ABCB1* (3435 C/T)**	**Tobacco users *N* = 43 (%)**	**Tobacco nonuser *N* = 88 (%)**	**p** **value**	**OR (95% CI)**
CC	7 (16.3%)	17 (19.3%)	—	1 (Reference)
CT	17 (39.5%)	33 (37.5%)	0.74	0.80 (0.28–2.46)
TT	19 (44.2%)	38 (43.2%)	0.98	1.006 (0.43–2.32)
**Genotype *M ABCB1* (1236 C/T)**	**Alcohol users *N* = 7 (%)**	**Alcohol nonusers *N* = 27 (%)**	**p** **value**	**OR (95% CI)**
**HIV-infected individuals with hepatotoxicity**				
CC	4 (57.1%)	12 (44.4%)	—	1 (Reference)
CT	1 (14.3%)	11 (40.7%)	0.33	0.29 (0.025–3.43)
TT	2 (28.6%)	4 (14.8%)	0.88	1.50 (0.13–17.35)
**Genotype *ABCB1* (3435 C/T)**	**Alcohol users *N* = 7 (%)**	**Alcohol nonusers *N* = 27 (%)**	**p** **value**	**OR (95% CI)**
CC	3 (42.9)	5 (18.5%)	—	1 (Reference)
CT	3 (42.9)	11 (40.7%)	0.63	0.60 (0.077–4.73)
TT	1 (14.3)	11 (40.7%)	0.32	0.26 (0.018–3.76)
**HIV-infected individuals without hepatotoxicity**				
**Genotype *ABCB1* (1236 C/T)**	**Alcohol users *N* = 44 (%)**	**Alcohol nonusers *N* = 87 (%)**	**p** **value**	**OR (95% CI)**
CC	23 (52.3%)	36 (41.4%)	—	1 (Reference)
CT	18 (40.9%)	38 (43.7%)	0.95	1.02 (0.45–2.35)
TT	3 (6.8%)	13 (14.9%)	0.20	0.40 (0.098–1.64)
**Genotype *ABCB1* (3435 C/T)**	**Alcohol users *N* = 44 (%)**	**Alcohol nonusers *N* = 87 (%)**	**p** **value**	**OR (95% CI)**
CC	6 (13.6%)	18 (20.7%)	—	1 (Reference)
CT	22 (50.0%)	28 (32.2%)	0.12	2.47 (0.79–7.70)
TT	16 (36.4%)	41 (47.1%)	0.81	1.15 (0.37–3.59)
**HIV-infected individuals with hepatotoxicity**				
**Genotype *ABCB1* (1236 C/T)**	**Nevirapine users *N* = 23 (%)**	**Efavirenz users *N* = 11 (%)**	**p** **value**	**OR (95% CI)**
CC	11 (47.8%)	5 (45.5%)	—	1 (Reference)
CT	7 (30.4%)	5 (45.5%)	0.64	0.69 (0.14–3.35)
TT	5 (21.7%)	1 (9.1%)	0.55	2.11 (0.18–24.66)
**Genotype *ABCB1* (3435 C/T)**	**Nevirapine users *N* = 23 (%)**	**Efavirenz users *N* = 11 (%)**	**p** **value**	**OR (95% CI)**
CC	7 (30.4%)	1 (9.1%)	—	1 (Reference)
CT	8 (34.8%)	6 (54.5%)	0.18	0.19 (0.017–2.11)
TT	8 (34.8%)	4 (36.4%)	0.43	0.37 (0.030–4.49)
**HIV-infected individuals without hepatotoxicity**				
**Genotype *ABCB1* (1236 C/T)**	**Nevirapine users *N* = 119 (%)**	**Efavirenz users *N* = 12 (%)**	**p** **value**	**OR (95% CI)**
CC	52 (43.7%)	7 (58.3%)	—	1 (Reference)
CT	52 (43.7%)	4 (33.3%)	0.45	1.66 (0.44–6.24)
TT	15 (12.6%)	1 (8.3%)	0.55	1.96 (0.22–17.42)
**Genotype *ABCB1* (3435 C/T)**	**Nevirapine users *N* = 119 (%)**	**Efavirenz users *N* = 12 (%)**	**p** **value**	**OR (95% CI)**
CC	24 (20.2%)	0 (0.0%)	NS	—
CT	44 (37.0%)	6 (50.0%)	—	1 (Reference)
TT	51 (42.9%)	6 (50.0%)	0.81	1.16 (0.34–3.12)
**Genotype *ABCB1* (1236 C/T)**	**Alcohol +Nevirapine users *N* = 5 (%)**	**Nevirapine users +** **alcohol nonuser *N* = 18 (%)**	**p** **value**	**OR (95% CI)**
**HIV-infected individuals with hepatotoxicity**				
CC	3 (60.0%)	8 (44.44%)	—	1 (Reference)
CT	0 (0.0%)	7 (38.89%)	NS	—
TT	2 (40.0%)	3 (16.67%)	0.55	2.21 (0.17–29.21)
**Genotype *ABCB1* (3435 C/T)**	**Alcohol +Nevirapine users *N* = 5 (%)**	**Nevirapine users + alcohol nonuser *N* = 18 (%)**	**p** **value**	**OR (95% CI)**
CC	3 (60.0%)	4 (22.22%)	—	1 (Reference)
CT	1 (20.0%)	7 (38.39%)	0.38	0.27 (0.015–5.01)
TT	1 (20.0%)	7 (38.39%)	0.75	0.61 (0.031–12.05)
**Genotype *ABCB1* (1236 C/T)**	**Alcohol + efavirenz users *N* = 2 (%)**	**Efavirenz users + alcohol nonusers *N* = 9 (%)**	**p** **value**	**OR (95% CI)**
CC	1 (50.0%)	4 (44.44%)	—	1 (Reference)
CT	1 (50.0%)	4 (44.44%)	0.85	0.73 (0.028–18.97)
TT	0 (0.0%)	1 (11.12%)	NS	—
**Genotype *ABCB1* (3435 C/T)**	**Alcohol + efavirenz users *N* = 2 (%)**	**Efavirenz users + alcohol nonusers *N* = 9 (%)**	**p** **value**	**OR (95% CI)**
CC	0 (0.0%)	1 (11.12%)	NS	—
CT	2 (100%)	4 (44.44%)	—	1 (Reference)
TT	0 (0.0%)	4 (44.44%)	NS	—
**HIV-infected individuals without hepatotoxicity**				
**Genotype *ABCB1* (1236 C/T)**	**Alcohol + nevirapine users *N* = 38 (%)**	**Nevirapine users + Alcohol nonusers *N* = 81 (%)**	**p** **value**	**OR (95% CI)**
CC	18 (47.37%)	34 (41.98%)	—	1 (Reference)
CT	17 (44.74%)	35 (43.20%)	0.68	1.20 (0.50–2.89)
TT	3 (7.89%)	12 (14.82%)	0.40	0.54 (0.13–2.26)
**Genotype *ABCB1* (3435 C/T)**	**Alcohol + nevirapine users *N* = 38 (%)**	**Nevirapine users + alcohol nonusers *N* = 81 (%)**	**p** **value**	**OR (95% CI)**
CC	6 (15.78%)	18 (22.22%)	—	1 (Reference)
CT	17 (44.74%)	27 (33.33%)	0.23	2.04 (0.64–6.53)
TT	15 (39.47%)	36 (44.45%)	0.74	1.22 (0.39–3.84)
**Genotype *ABCB1* (1236 C/T)**	**Alcohol + efavirenz users *N* = 6 (%)**	**Efavirenz users + alcohol nonuser *N* = 6 (%)**	**p** **value**	**OR (95% CI)**
CC	5 (83.33%)	2 (33.33%)	—	1 (Reference)
CT	1 (16.67%)	3 (50.0%)	0.16	0.13 (0.008–2.18)
TT	0 (0.0%)	1 (16.67%)	NS	—
**Genotype *ABCB1* (3435 C/T)**	**Alcohol + efavirenz users *N* = 6 (%)**	**Efavirenz users + alcohol nonuser *N* = 6 (%)**	**p** **value**	**OR (95% CI)**
CC	0 (0.0%)	0	NS	—
CT	5 (83.33%)	1 (16.67%)	—	1 (Reference)
TT	1 (16.67%)	5 (83.33%)	**0.04**	0.04 (0.002–0.83)

*Note: N* = number of subjects, % = frequency of subjects, OR, and 95% CIs were derived from the logistic regression model comparing the homozygous wild‐type genotype/allele (CC genotype and C allele were used as a reference for *ABCB1* 1236CT, 1236TT genotypes and 1236 T allele and 3435CT, 3435TT genotypes and 3435T allele) with other genotypes/alleles. Significant *p* values are shown in bold​ (*p* < 0.05).

Abbreviation: NS, not significant.

Although none of these associations reached statistical significance, the data suggest potential gene–environment–drug interactions that should be explored further. Given the cross‐sectional design and small subgroup sizes, causality cannot be inferred.

## 4. Discussion

This is the first study from India investigating ABCB1 polymorphisms in the context of ARV‐associated hepatotoxicity. Many functionally related processes, such as transcription, splicing, cotranslational folding, and mRNA stability, can be affected by a synonymous variation [31]. In addition, synonymous single‐nucleotide variants (sSNVs) affect transcriptional regulatory factors and splicing into the protein‐coding region, thereby controlling gene expression [31, 32]. Through entropy‐induced changes in mRNA dynamics, synonymous functional variations close to the translation start site affect translation efficiency [33]. The formation of secondary and tertiary protein structures is strongly correlated with cotranslational folding; alpha‐helix formation can occur in the ribosome tunnel, whereas tertiary structure formation can take place before the protein has fully exited the ribosome [34]. Low concentrations of tRNA can cause rare codon variations of frequently occurring synonymous codons to decrease the translation rate, slow or stop the elongation of the peptide chain, and affect cofolding [8–35]. The *ABCB1* gene encodes an ATP‐dependent membrane efflux transporter, and P‐gps are the substrate for the genetic variation that affects a patient’s response to drugs. The expression of *ABCB1* varies among populations [36].

The prevalence of *ABCB1* 3435 C/T polymorphism in our healthy control population was similar to studies carried out in European, North Indian, Turkish, and Asian populations [21–23, 26, 37], but differed from studies conducted in people from China, Iran, and Thailand [21, 28]. Moreover, the prevalence of the *ABCB1* 1236 C/T polymorphism in our healthy control population was similar to the prevalence found in the study conducted in the North Indian population [38]; however, it differs from that found in the populations of South Africans, Chinese, and Mexicans [20, 39]. In this study, *ABCB1* 1236 C/T and 3435 C/T polymorphisms were not significantly different among HIV‐infected individuals with and without hepatotoxicity and healthy controls.

In a genotype–phenotype analysis, the *ABCB1* 1236TT genotype showed a risk for severe hepatotoxicity (OR = 1.37, *p* = 0.57). However, the risk could not reach statistical significance because of the smaller number of patients in the hepatotoxicity group. The low phenotypic expression is determined by natural polymorphisms within P‐gp. Individuals with *the ABCB1 3435*TT genotype expressed lower levels of P‐gp, while those with a CC or CT genotype had higher levels of P‐gp. *The ABCB1* 3435T allele was associated with a decreased risk of hepatotoxicity during combination ART that included NVP [21]. Furthermore, the *ABCB1* 3435 C/T polymorphism was associated with a lower risk of hepatotoxicity with NNRTI‐containing regimens [22, 40].

In complex diseases, where more than one locus contributes to disease susceptibility, instead of single‐locus polymorphisms, haplotypes are potentially more important as different combinations of alleles in the different genes may have different effects on gene expression [41]. In this study, we evaluated the relationship between ABCB1 haplotypes and the risk of ARV‐induced hepatotoxicity. We found that the TT and CC haplotypes were associated with reduced hepatotoxicity severity, suggesting a potential protective effect. Meanwhile, the TC haplotype showed a trend toward increased hepatotoxicity severity (*p* = 0.06), though not statistically significant. However, this association did not reach statistical significance and therefore must be interpreted cautiously. The wide CI indicates uncertainty, likely due to the relatively small number of cases in the hepatotoxicity group. These associations should be interpreted with caution given the study’s limited sample size and lack of plasma drug concentration data.

Also, we analyzed the effect of *ABCB1* polymorphisms on the advancement of HIV disease. The current CD4 count is considered a substitute marker for HIV‐1 infection. The results could be influenced by the duration of HIV infection because the duration of HIV infection is not known. In our study, the *ABCB1* 1236 C/T and 3435 C/T polymorphisms did not differ significantly between different stages of HIV infection and healthy controls. *ABCB1* 1236CT, 1236TT, and 3435CT genotypes were associated with AIDS progression (*p* = 0.024, *p* = 0.026, and *p* = 0.005, respectively) [42]. In addition, individuals with the 3435 TT genotype had higher CD4+ levels after six months of therapy [5].

Gene–environment interaction defines the etiology of a disease [43]. A case‐only method is preferred over a case–control method for evaluating gene–environment interactions. The selection criteria for the study of environmental influences on cases must be matched with the controls in the population [44]. As a result, the case‐only method was adopted in this study to investigate the relationship between gene polymorphism and tobacco, alcohol, and drug use. A study suggested that heavy alcohol consumption had a negative impact on the CD4 cell count in HIV patients lacking ARV treatment [45]. The ART response is decreased among HIV‐infected women who are smokers [46]. In our study, the *ABCB1* 3435CT genotype in the presence of alcohol was not likely to be associated with HIV disease progression (OR = 2.47, *p* = 0.12) when compared between alcohol users and nonusers among HIV‐infected individuals without hepatotoxicity. It supports the idea that individuals with *ABCB1* 3435CT genotypes are more susceptible to alcohol‐related hepatotoxicity.

*In* HIV‐infected individuals with hepatotoxicity, the *ABCB1* 1236TT genotype along with NVP usage was not likely to be associated with the risk for hepatotoxicity severity (OR = 2.11, *p* = 0.55). Similarly, the ABCB1 1236CT and 1236TT in the presence of NVP genotypes revealed a risk for acquisition of hepatotoxicity (OR = 1.66, *p* = 0.45; OR = 1.96, *p* = 0.55), but risk could not reach statistical significance when compared between NVP users and nonusers among HIV‐infected individuals without hepatotoxicity. This suggests that individuals with the *ABCB1* 1236TT genotype, along with NVP, may have a greater susceptibility to drug‐related hepatotoxicity.

In HIV‐infected individuals with hepatotoxicity, the *ABCB1* 1236TT genotype in the presence of alcohol and NVP was not likely to be associated with a risk for the severity of hepatotoxicity (OR = 2.21, *p* = 0.55). Similarly, in HIV‐infected individuals without hepatotoxicity, the *ABCB1* 3435CT genotype, along with NVP and alcohol usage, was not likely to be associated with a risk for the acquisition of hepatotoxicity (OR = 2.04, *p* = 0.23). It suggests that in the presence of alcohol and NVP with *ABCB1* 3435CT and 1236TT genotypes, the individuals are more prone to develop alcohol and drug‐induced hepatotoxicity and its severity, respectively. The *ABCB1* 1236T and 1235T alleles lead to a reduced NNRTI plasma concentration, affecting the virological response to HAART [47]. Haas et al. assessed the association of the two *ABCB1* variants with outcome responses in patients receiving EFV and suggested no significant association with plasma EFV concentrations [21].

This study has limitations; for instance, it could only assess association and not decide causality. Initially, we allocated a ratio of 1:4 for case controls. However, we were unable to manage matched enrollment in the controls. Despite this, our case–control ratio is approximately 1:3, which may be sufficient for this study. In addition, we did not assess the plasma drug concentration in our subject participants. Hence, we were not able to address the correlation of *ABCB1* polymorphisms with plasma drug levels in HIV patients.

In conclusion, ABCB1 1236 C/T and 3435 C/T polymorphisms were not significantly associated with hepatotoxicity in HIV‐infected individuals. However, certain haplotypes may influence the severity of ARV‐induced hepatotoxicity. No significant gene–environment interactions were observed with alcohol, tobacco, or NVP use. Given ABCB1’s role in drug transport and variability in expression across ethnic groups, further studies involving larger cohorts and including other transporter (e.g., MRP4) and metabolizing enzyme genes (e.g., CYP450s, GST) are warranted to better understand genetic influences on drug clearance and treatment outcomes.

**Nomenclature**
HIV: Human immunodeficiency virus
ABCB1: ATP‐binding cassette subfamily B member 1
NNRTIs: Non‐nucleoside reverse‐transcriptase inhibitors
ARV: Antiretroviral
*ART*
: Antiretroviral therapy
NVP: Nevirapine
EFV: Efavirenz
SNP: Single‐nucleotide polymorphism
PCR–RFLP: Polymerase chain reaction—restriction fragment length polymorphisms
ADR: Adverse drug reaction
LFT: Liver function test
SGOT: Serum glutamic oxaloacetic transaminase
SGPT: Serum glutamic pyruvic transaminase
ELISA: Enzyme‐linked immunosorbent assay
ABC: ATP‐binding cassette.

## Ethics Statement

The study is approved by the Institutional Ethics Committee (IEC): NARI/EC/ICF, Version 1.0, dated August 28, 2013. No animals were used for the studies that formed the basis of this research. All human procedures followed were in accordance with the guidelines of the Helsinki Declaration of 1975.

## Consent

Written informed consent was taken for publication.

## Disclosure

The earlier version of this manuscript is present in Research Square (https://doi.org/10.21203/rs.3.rs-51053/v1) and titled Analysis of MDR1 Polymorphisms Among HIV‐Infected Individuals on ARV Therapy from Western India & HariOm Singh, Dharmesh Samani. MDR1 Polymorphisms and Modulation of ARV Drug Induced Hepatotoxicity, 08 November 2021, PREPRINT [Version 1 available at Research Square (https://doi.org/10.21203/rs.3.rs-1019782/v1)].

## Conflicts of Interest

The authors declare no conflicts of interest.

## Author Contributions

HariOm Singh: overall supervision.

Dharmesh Samani: experimental work.

Supriya D. Mahajan: data analysis.

## Funding

No funding was received for this research.

## Data Availability

The datasets used and/or analyzed during the current study are available from the corresponding author on reasonable request.
